# Social Media Misinformation, Contraceptive Literacy, and Psychological Well-Being Among Romanian Adolescents and Young Adults

**DOI:** 10.3390/healthcare14131836

**Published:** 2026-06-24

**Authors:** Denisa Hinoveanu, Ahmed Abu-Awwad, Simona-Alina Abu-Awwad, Anca-Mihaela Bînă, Lavinia Stelea, Adrian Gluhovschi, Daniela Gurguș

**Affiliations:** 1 Doctoral School, “Victor Babes” University of Medicine and Pharmacy, 300041 Timisoara, Romania; adriana.hinoveanu@umft.ro; 2 “Pius Brinzeu” Emergency Clinical County Hospital, 300723 Timisoara, Romania; ahm.abuawwad@umft.ro (A.A.-A.); lungu.anca@umft.ro (A.-M.B.); stelea.lavinia@umft.ro (L.S.); gluhovschi.adrian@umft.ro (A.G.); 3 Centre for Translational Research and Systems Medicine, “Victor Babes” University of Medicine and Pharmacy, 300041 Timisoara, Romania; 4 Department XV-Discipline of Orthopedics-Traumatology, “Victor Babes” University of Medicine and Pharmacy, 300041 Timisoara, Romania; 5 Department of Obstetrics and Gynecology, “Victor Babes” University of Medicine and Pharmacy, 300041 Timisoara, Romania; 6 Department of Balneology, Medical Recovery and Rheumatology, Family Discipline, Center for Preventive Medicine, “Victor Babeș” University of Medicine and Pharmacy, 300041 Timisoara, Romania

**Keywords:** adolescents, contraception, social media, misinformation, contraceptive literacy, digital health literacy, anxiety, reproductive health, mental health, TikTok

## Abstract

**Highlights:**

**What are the main findings?**

- Social media represented the primary source of contraceptive information for most Romanian adolescents and young adults and was associated with lower contraceptive knowledge scores.
- Higher exposure to contraceptive misinformation was independently associated with increased anxiety symptoms, poorer contraceptive literacy, and greater endorsement of infertility-related fears.

**What are the implications of the main findings?**

- Digital health literacy and prior gynecological counseling may act as protective factors against misinformation and inadequate contraceptive knowledge.
- Integrating evidence-based reproductive health education into social media environments may improve contraceptive literacy and support adolescent psychological well-being.

**Abstract:**

**Background/Objectives:** The rapid expansion of social media platforms has profoundly changed the way adolescents access reproductive health information. While digital environments increase accessibility to contraceptive content, they also facilitate the dissemination of misinformation, potentially influencing both contraceptive literacy and psychological well-being. The present study aimed to evaluate the relationship between sources of contraceptive information, contraceptive misinformation endorsement, contraceptive knowledge, and mental health indicators among Romanian adolescents and young adults. **Methods:** A cross-sectional observational study was conducted in a cohort of 210 Romanian adolescents and young adults. Participants completed a structured self-administered questionnaire assessing demographic characteristics, contraceptive information sources, digital health behaviors, contraceptive misconceptions, and contraceptive knowledge. Anxiety and depressive symptoms were evaluated using the Generalized Anxiety Disorder-7 (GAD-7) and Patient Health Questionnaire-9 (PHQ-9) scales. Correlation analyses and multivariable logistic regression models were performed to identify factors associated with poor contraceptive knowledge and moderate-to-severe anxiety. **Results:** Social media represented the primary source of contraceptive information for 58.1% of participants. Individuals relying predominantly on social media demonstrated significantly lower contraceptive knowledge questionnaire (CKQ) scores compared to those obtaining information from healthcare professionals (5.9 ± 1.8 vs. 8.1 ± 1.7, *p* < 0.001). Contraceptive misinformation endorsement was inversely correlated with CKQ scores (r = −0.44, *p* < 0.001) and positively associated with anxiety (r = 0.47, *p* < 0.001) and depressive symptoms (r = 0.41, *p* < 0.001). In multivariable analyses, primary reliance on social media (OR 2.21, 95% CI 1.12–4.34, *p* = 0.022) and low digital health literacy (OR 2.94, 95% CI 1.51–5.71, *p* = 0.001) were independently associated with poor contraceptive knowledge. Higher misinformation endorsement, infertility-related fears, and high social media exposure were independently associated with moderate-to-severe anxiety. **Conclusions:** Contraceptive misinformation endorsement was associated with lower contraceptive literacy and poorer psychological outcomes among adolescents and young adults. These findings highlight the growing importance of digital health literacy. However, given the cross-sectional design, the observed relationships should be interpreted as associations rather than causal effects, and longitudinal studies are required to clarify their directionality.

## 1. Introduction

In recent years, access to information regarding sexual and reproductive health has undergone a profound transformation, largely driven by the rapid expansion of digital platforms and social media [1,2]. For adolescents and young women, traditional sources of information (such as school-based education or medical counseling) are increasingly being supplemented, and sometimes replaced, by online content [3,4]. While this shift has improved accessibility and exposure to health-related topics, it has also introduced a new layer of complexity: the widespread dissemination of incomplete, misleading, or inaccurate information, particularly in the domain of contraception [5,6,7].

Adolescence represents a critical period for the development of sexual identity, autonomy, and decision-making skills [8]. During this stage, individuals are particularly vulnerable to external influences, including peer norms and digital content, due to ongoing neurocognitive maturation and heightened sensitivity to social validation [9,10]. As a result, information encountered online may significantly shape perceptions, attitudes, and behaviors related to contraceptive use. Importantly, the ability to critically evaluate the credibility of such information, commonly referred to as health literacy, remains variable and often underdeveloped in this population [11].

Recent studies have highlighted the growing role of social media platforms, such as TikTok and Instagram, as primary sources of health information among adolescents [2,12]. However, the content shared on these platforms is not consistently evidence-based and frequently includes personal anecdotes, subjective experiences, or exaggerated claims regarding contraceptive methods [5]. Common themes include fears related to infertility, hormonal imbalance, or long-term health consequences, which are often presented without scientific context. These narratives, although compelling, may contribute to the persistence of contraceptive myths and influence decision-making in a way that increases the risk of inconsistent or incorrect contraceptive use [7,13].

At the same time, there is increasing recognition of the bidirectional relationship between contraceptive knowledge and mental well-being [14,15]. Limited or inaccurate knowledge may contribute to uncertainty, anxiety, and reduced perceived control over reproductive health, while psychological distress itself may impair the ability to seek, interpret, and apply reliable information [16]. This interaction is particularly relevant in adolescents, where both cognitive and emotional regulation processes are still developing.

Despite the growing body of international literature on digital health information and adolescent behavior, data from Eastern European countries, including Romania, remain limited [17]. Cultural factors, variability in sexual education programs, and differences in healthcare accessibility may further influence how adolescents obtain and interpret contraceptive information [18,19]. Understanding these dynamics is essential for developing targeted educational strategies that address both informational gaps and the broader psychosocial context in which reproductive decisions are made [20].

Therefore, the present study aims to explore the sources of contraceptive information among Romanian adolescents and to assess the prevalence of misinformation and its association with contraceptive knowledge and mental health indicators. By identifying patterns in information acquisition and their potential impact, this research seeks to contribute to the development of more effective, evidence-based interventions tailored to the needs of this population.

## 2. Materials and Methods

### 2.1. Study Design and Population

The present study was designed as a cross-sectional observational analysis aimed at exploring the relationship between sources of contraceptive information, the prevalence of misinformation, and their association with both contraceptive knowledge and mental health indicators in a cohort of Romanian adolescents and young adults. Rather than focusing strictly on information exposure, the study was structured to capture how this information is understood and integrated into personal decision-making, reflecting a more nuanced, real-world perspective on reproductive health literacy [21].

A total of 210 participants were included in the final analysis, with a mean age of approximately 19–20 years, representing a population already partially engaged in reproductive health decision-making. Data collection was conducted between October 2021 and June 2024, in parallel with the data collection framework of the doctoral research project from which the present analysis was derived. Participation was voluntary and anonymous, and all individuals provided informed consent prior to inclusion. Questionnaires with incomplete or inconsistent responses were excluded to ensure data reliability.

Participants were recruited using convenience sampling through online distribution of the questionnaire via social media platforms, university student groups, and peer-sharing networks.

Inclusion criteria consisted of Romanian adolescents and young adults aged between 16 and 24 years who were able to understand and complete the questionnaire in Romanian and who voluntarily agreed to participate in the study. Participants were required to reside in Romania during the study period and to provide informed consent prior to inclusion. For participants younger than 18 years, electronic informed assent was obtained together with parental or guardian acknowledgment in accordance with institutional ethical recommendations for anonymous questionnaire-based research involving minors.

Exclusion criteria included incomplete questionnaires, duplicate submissions, inconsistent response patterns, questionnaires completed in unrealistically short time intervals, and responses containing contradictory demographic or behavioral information. Participants reporting a previously diagnosed severe psychiatric disorder requiring recent hospitalization or acute psychiatric intervention were also excluded in order to reduce potential confounding effects on the assessment of mental health indicators and contraceptive-related perceptions. Additional exclusion criteria included inability to understand the questionnaire language, refusal to provide consent, and questionnaires with extensive missing data affecting the primary study variables.

To minimize the risk of duplicate participation and improve data reliability, several preventive measures were implemented during online data collection. The survey platform was configured, whenever technically feasible, to limit multiple submissions from the same device and browser session. In addition, questionnaires with highly similar demographic profiles, identical response patterns, or unrealistically short completion times were manually screened and excluded when duplicate participation was suspected.

To reduce the likelihood of duplicate participation, the online survey platform was configured to limit multiple submissions from the same device and browser session whenever technically feasible.

### 2.2. Data Collection and Variables

Data were collected using a structured, self-administered questionnaire designed to capture both behavioral patterns and cognitive aspects related to contraceptive use. Basic demographic and lifestyle characteristics were recorded, including age, residence, sexual activity, prior contraceptive use, smoking, and alcohol consumption, allowing for contextual interpretation of the findings.

A central component of the questionnaire focused on identifying the primary source of contraceptive information. Participants were asked to indicate whether their knowledge was mainly derived from social media platforms, school-based education, healthcare professionals, or informal networks such as family and peers. This distinction was particularly important, given the variability in the quality and reliability of information across these channels.

In addition to information sources, the study assessed the level of contraceptive knowledge through a composite score based on multiple items addressing key aspects such as mechanism of action, effectiveness, and safety of commonly used methods. Parallel to this, the presence of misinformation was evaluated using targeted questions reflecting frequently encountered misconceptions, including beliefs related to infertility, long-term health effects, or the mechanism of emergency contraception. These items were selected based on patterns consistently observed in both the doctoral dataset and the existing literature [22].

The contraceptive knowledge questionnaire (CKQ) consisted of 10 multiple-choice items designed to assess essential aspects of contraceptive literacy, including mechanism of action, effectiveness, safety profile, appropriate use, and common misconceptions related to modern contraceptive methods. The Contraceptive Knowledge Questionnaire (CKQ) was specifically developed for the purposes of the present study based on a review of the contemporary literature addressing contraceptive literacy, adolescent reproductive health education, and commonly reported contraceptive misconceptions. The initial item pool was generated by a multidisciplinary team consisting of gynecologists, public health researchers, and reproductive health educators. Content validity was assessed through expert review to ensure that the selected items adequately covered key domains of contraceptive knowledge, including mechanism of action, effectiveness, safety profile, appropriate use, emergency contraception, and fertility-related misconceptions. Prior to large-scale administration, the questionnaire was pilot-tested in a sample of 15 adolescents and young adults representative of the target population, resulting in minor modifications to item wording and clarity.

Each correct answer was assigned 1 point, while incorrect or “I don’t know” responses received 0 points, generating a total score ranging from 0 to 10, with higher scores indicating better contraceptive knowledge. Because the CKQ was designed as a knowledge assessment tool rather than a diagnostic instrument, all items contributed equally to the total score. Internal consistency was evaluated using Cronbach’s alpha coefficient, which demonstrated acceptable reliability (Cronbach’s α = 0.78). Although the questionnaire has not undergone formal external validation, its development process, expert review, pilot testing, and internal consistency analysis support its suitability for exploratory assessment of contraceptive knowledge in this population.

Contraceptive misinformation endorsement was assessed using a dedicated composite score consisting of eight items reflecting common contraceptive misconceptions frequently encountered in social media and digital health environments. Rather than measuring direct exposure to misinformation, the instrument was designed to evaluate the extent to which participants endorsed misinformation-consistent beliefs related to contraception. The items addressed misconceptions regarding contraceptive safety, effectiveness, fertility, emergency contraception, and potential long-term health effects.

The misinformation items were developed following a review of contemporary literature addressing contraceptive myths, reproductive health misinformation, and social media-related health communication among adolescents and young adults. Content relevance and clarity were reviewed by a multidisciplinary team including gynecologists and public health researchers. Prior to administration, the items were pilot-tested in a small sample representative of the target population, resulting in minor wording adjustments to improve comprehensibility and reduce ambiguity.

Because the instrument assessed endorsement of misinformation-related beliefs rather than objectively verified exposure to misinformation, the resulting score should be interpreted as a measure of misinformation endorsement. Direct exposure to misinformation was not quantified, and participants may have acquired these beliefs through multiple sources, including social media, peers, family members, or other information channels.

Participants indicated agreement or disagreement with each statement. Each misinformation-consistent response was assigned 1 point, generating a total misinformation endorsement score ranging from 0 to 8, with higher scores indicating greater endorsement of contraceptive misinformation.

Examples of misinformation items included statements such as: “Hormonal contraception causes permanent infertility”, “Emergency contraception is equivalent to abortion”, “Natural contraceptive methods are as effective as modern contraceptive methods”, and “Hormonal contraceptives inevitably cause severe long-term health damage”. These items were selected because they represent recurrent misconceptions consistently identified in studies evaluating contraceptive misinformation among adolescents and young adults and are frequently encountered in social media environments. All items contributed equally to the composite misinformation score because the instrument was designed as an exploratory measure of misinformation endorsement rather than a weighted psychometric scale.

Digital health literacy was assessed using a structured self-reported questionnaire adapted from previously published digital health literacy frameworks for adolescents and young adults [23]. The instrument consisted of 8 items evaluating the participants’ perceived ability to search for, understand, critically evaluate, and apply online health-related information, particularly information related to contraception and reproductive health obtained from social media platforms and internet-based sources.

The instrument was adapted from previously published digital health literacy frameworks developed for adolescents and young adults and was modified to reflect the contemporary digital environments most frequently used by Romanian adolescents, including social media platforms commonly accessed for reproductive health information. The adaptation process involved linguistic refinement, contextual modification of selected items, and review by researchers with expertise in adolescent health and reproductive medicine to ensure content relevance and comprehensibility for the target population.

Each item was evaluated using a 5-point Likert scale ranging from 1 (“very difficult”) to 5 (“very easy”), generating a cumulative score ranging from 8 to 40 points. Higher scores reflected better perceived digital health literacy. Based on the total score distribution, participants were categorized into three predefined groups: low digital health literacy (8–18 points), moderate digital health literacy (19–29 points), and high digital health literacy (30–40 points).

All items contributed equally to the total score. Higher scores reflected greater perceived ability to identify, access, understand, critically evaluate, and apply online health information. Because the instrument was intended to provide an overall assessment of digital health literacy rather than to evaluate individual competency domains separately, no differential weighting of items was applied.

Prior to administration, the adapted questionnaire underwent pilot testing in a small sample representative of the target population to evaluate clarity, readability, and item interpretation. Although formal psychometric validation was beyond the scope of the present study, the use of an established conceptual framework, expert review, and pilot testing support the suitability of the instrument for exploratory assessment of digital health literacy.

The questionnaire was adapted to reflect contemporary digital media environments frequently used by Romanian adolescents and young adults, including social media-based health information exposure. Internal consistency analysis demonstrated acceptable reliability for the digital health literacy scale (Cronbach’s α = 0.81).

For regression analysis, low contraceptive knowledge was defined as a CKQ score below the cohort median (CKQ < 7). Median-based dichotomization was chosen to facilitate the identification of factors associated with relatively lower knowledge levels within the study population and to improve the interpretability of the logistic regression models.

### 2.3. Assessment of Mental Health Indicators

Recognizing the growing evidence supporting a link between reproductive health literacy and psychological well-being, mental health status was assessed using validated screening tools. Anxiety symptoms were evaluated using the Generalized Anxiety Disorder-7 (GAD-7) scale [24], while depressive symptoms were measured with the Patient Health Questionnaire-9 (PHQ-9) [25]. Both instruments are widely used in clinical and research settings and provide reliable quantification of symptom severity, particularly in young populations. A GAD-7 score ≥ 10 was considered indicative of moderate-to-severe anxiety, in accordance with previously validated cut-off values commonly used in adolescent and young adult populations [24]. This threshold was selected because it represents the most commonly validated cut-off for clinically relevant anxiety symptoms in adolescent and young adult populations.

This approach allowed for the simultaneous analysis of cognitive (knowledge-based) and emotional (mental health) dimensions, offering a more integrated understanding of how adolescents relate to contraceptive information.

### 2.4. Statistical Analysis

Statistical analysis was performed using IBM SPSS Statistics version 29.0 (IBM Corp., Armonk, NY, USA). Continuous variables were expressed as mean ± standard deviation (SD), while categorical variables were summarized as frequencies and percentages.

The distribution of continuous variables was assessed using the Shapiro–Wilk test. Comparisons between two independent groups were performed using the independent samples *t*-test for normally distributed variables. Associations between categorical variables were evaluated using the chi-square test.

Prior to large-scale distribution, the questionnaire was pilot-tested on a small group of 15 university students and late adolescents representative of the target population in order to evaluate clarity, comprehensibility, wording consistency, and completion time. Minor linguistic and structural adjustments were subsequently performed to improve readability and reduce ambiguity of selected items before final administration.

Correlations between contraceptive knowledge scores (CKQ), misinformation endorsement, and mental health indicators (GAD-7 and PHQ-9 scores) were assessed using Pearson correlation coefficients. Correlation strength was interpreted according to standard recommendations.

To further explore independent predictors of poor contraceptive knowledge and psychological distress, multivariable logistic regression analyses were performed using the enter method. For the first regression model, low contraceptive knowledge was used as the dependent variable, defined as a CKQ score below the cohort median (CKQ < 7). Independent variables included primary reliance on social media, predominant TikTok use, low digital health literacy, absence of prior gynecological counseling, urban/rural residence, socioeconomic status, and medical-related education.

For the second regression model, moderate-to-severe anxiety was used as the dependent variable, defined as a GAD-7 score ≥ 10. Independent variables included misinformation endorsement, low contraceptive knowledge, high health-related social media exposure (>3 h/day), infertility-related contraceptive fear, predominant TikTok use, and digital health literacy level.

Variables included in the multivariable logistic regression models were selected a priori based on clinical relevance, theoretical plausibility, and previously reported associations in the literature. Emphasis was placed on factors potentially influencing contraceptive knowledge acquisition, misinformation endorsement, digital health literacy, and psychological well-being among adolescents and young adults. Odds ratios (ORs) with 95% confidence intervals (CIs) were calculated for all regression analyses. Prior to model construction, multicollinearity among predictor variables was assessed using variance inflation factors (VIFs). No evidence of problematic multicollinearity was identified, with all VIF values remaining below commonly accepted thresholds. Model calibration was evaluated using the Hosmer-Lemeshow goodness-of-fit test, and model discrimination was assessed using the area under the receiver operating characteristic curve (AUC). A two-tailed *p*-value < 0.05 was considered statistically significant throughout the study.

The selected variables were chosen to represent demographic, educational, behavioral, and digital health-related domains that could plausibly influence contraceptive knowledge acquisition and psychological well-being while avoiding excessive model complexity relative to the available sample size.

Given the exploratory observational design of the study, the sample size was estimated pragmatically based on feasibility and expected recruitment capacity during the study period. However, an a priori estimation suggested that a sample of approximately 180–200 participants would provide adequate statistical power (80%) to detect moderate effect sizes (Cohen’s d ≈ 0.5) for the primary comparative analyses at a significance level of α = 0.05. The final study cohort of 210 participants was therefore considered adequate for the planned analyses.

### 2.5. Ethical Considerations

The study was conducted in accordance with the ethical principles outlined in the Declaration of Helsinki. Participation was entirely voluntary, and anonymity was ensured throughout the data collection process. The use of a self-administered questionnaire was intended to reduce reporting bias, particularly in relation to sensitive topics such as sexual behavior and mental health. Data confidentiality was maintained in compliance with current data protection regulations.

The study protocol was approved by the Local Research Ethics Committee of the Victor Babeș University of Medicine and Pharmacy, Timișoara, Romania (Approval No. 27/2021 in 10 January 2021 and No. 25 on 9 January 2023), and was conducted in accordance with the principles of the Declaration of Helsinki.

## 3. Results

A total of 210 Romanian adolescents and young adults were included in the final analysis. The study population was characterized not only in terms of sociodemographic and reproductive health variables, but also according to patterns of digital information consumption and contraceptive-related behaviors. Particular attention was given to the participants’ primary sources of contraceptive information, use of social media platforms, prior medical counseling, and digital health literacy, in order to better understand the broader informational environment influencing reproductive health decision-making. The main baseline characteristics of the cohort are summarized in Table 1. The cohort was predominantly composed of university students (69.5%), urban residents (62.4%), and individuals reporting a moderate socioeconomic status (62.9%). These characteristics should be considered when interpreting the representativeness of the sample and the generalizability of the study findings to broader adolescent and young adult populations.

When exploring how participants access contraceptive information, a clear pattern emerged. Social media platforms represented the dominant source, reported by more than half of the respondents. In contrast, structured sources such as school-based education and healthcare professionals were significantly less frequently mentioned. This imbalance becomes particularly relevant when considering the variability in the quality of information available across these channels. The distribution of information sources is illustrated in Table 2.

Participants relying predominantly on social media demonstrated significantly lower contraceptive knowledge scores compared to those obtaining information primarily from healthcare professionals (5.9 ± 1.8 vs. 8.1 ± 1.7, *p* < 0.001). Similarly, participants using TikTok as their primary digital platform had lower mean CKQ scores compared to participants using other platforms (5.8 ± 1.9 vs. 7.4 ± 2.0, *p* = 0.002). In contrast, previous gynecological counseling was associated with significantly higher contraceptive knowledge levels (7.9 ± 1.8 vs. 6.2 ± 2.0, *p* < 0.001).

Beyond access to information, the analysis revealed a substantial persistence of misconceptions regarding contraceptive methods. A notable proportion of participants endorsed at least one incorrect belief, most frequently related to the long-term effects of hormonal contraception. Concerns regarding infertility, exaggerated health risks, or the perceived superiority of natural methods were commonly reported. These findings are summarized in Table 3.

Exposure to contraceptive misinformation demonstrated a moderate inverse correlation with CKQ scores (r = −0.44, *p* < 0.001), indicating that higher levels of misinformation endorsement were associated with poorer contraceptive literacy. Participants endorsing infertility-related misconceptions also reported significantly higher anxiety scores compared to those without such beliefs (GAD-7: 8.6 ± 4.5 vs. 5.9 ± 3.8, *p* = 0.001).

A similar pattern emerged when examining psychological outcomes. Lower levels of contraceptive knowledge were associated with higher levels of anxiety and depressive symptoms. Conversely, participants with higher knowledge scores reported more favorable mental health indicators. These relationships are summarized in Table 4.

Lower contraceptive knowledge scores were moderately associated with higher anxiety symptoms (r = −0.39, *p* < 0.001) and depressive symptoms (r = −0.34, *p* < 0.001). Similarly, misinformation endorsement demonstrated positive correlations with both GAD-7 scores (r = 0.47, *p* < 0.001) and PHQ-9 scores (r = 0.41, *p* < 0.001). Participants with low contraceptive knowledge (CKQ < 7) showed significantly higher mean anxiety scores compared to participants with adequate knowledge (8.4 ± 4.2 vs. 5.8 ± 3.7, *p* < 0.001). A similar trend was observed for depressive symptoms (PHQ-9: 7.5 ± 4.3 vs. 5.2 ± 3.6, *p* = 0.002).

A multivariable logistic regression analysis was performed to identify independent predictors of poor contraceptive knowledge. Primary reliance on social media was independently associated with increased odds of low contraceptive knowledge (OR 2.48, 95% CI 1.31–4.69, *p* = 0.005). Similarly, predominant TikTok use and low digital health literacy were significant predictors of inadequate contraceptive knowledge.

Participants without prior gynecological counseling also demonstrated significantly higher odds of poor contraceptive literacy. In contrast, medical-related education appeared to have a protective effect. Rural residence and low socioeconomic status showed trends toward lower knowledge levels, although these associations did not reach statistical significance. The complete regression model is presented in Table 5.

After adjustment for demographic, educational, and digital behavioral variables, primary reliance on social media remained independently associated with increased odds of low contraceptive knowledge (OR 2.21, 95% CI 1.12–4.34, *p* = 0.022). Low digital health literacy represented the strongest independent predictor of poor contraceptive literacy (OR 2.94, 95% CI 1.51–5.71, *p* = 0.001). Non-medical educational background also remained significantly associated with inadequate contraceptive knowledge.

In contrast, predominant TikTok use and low socioeconomic status showed positive associations with poor contraceptive literacy, although these relationships did not remain statistically significant after multivariable adjustment. Similarly, absence of prior gynecological counseling demonstrated a borderline association with low contraceptive knowledge (*p* = 0.058), suggesting a potential protective role of professional reproductive health counseling that may warrant further investigation in larger cohorts. To further explore the psychological dimension of contraceptive misinformation, a second multivariable logistic regression model was performed using moderate-to-severe anxiety as the dependent variable, defined as a GAD-7 score ≥ 10. After adjustment for digital behavior, contraceptive knowledge, misinformation endorsement, contraceptive-related fears, and digital health literacy, several independent predictors of moderate-to-severe anxiety were identified.

Higher misinformation endorsement was independently associated with increased odds of moderate-to-severe anxiety (OR 2.36, 95% CI 1.28–4.35, *p* = 0.006). Low contraceptive knowledge also remained a significant predictor of anxiety symptoms (OR 2.11, 95% CI 1.14–3.91, *p* = 0.018). Participants reporting infertility-related fear had more than two-fold higher odds of moderate-to-severe anxiety compared with those without this concern (OR 2.54, 95% CI 1.32–4.88, *p* = 0.005). High social media exposure, defined as more than 3 h/day of health-related social media use, was also significantly associated with anxiety (OR 1.98, 95% CI 1.04–3.76, *p* = 0.037). Predominant TikTok use showed a positive but borderline association, while low digital health literacy remained a significant independent predictor. The complete regression model is presented in Table 6.

In this model, misinformation endorsement, low contraceptive knowledge, infertility-related fear, high health-related social media exposure, and low digital health literacy were independently associated with moderate-to-severe anxiety. Predominant TikTok use showed a positive but borderline association, suggesting that platform-specific exposure may contribute to psychological vulnerability, although this effect was partially attenuated after adjustment for misinformation endorsement and digital health literacy. This finding supports the hypothesis that the psychological impact of social media may be mediated not only by platform use itself, but also by the quality of the information consumed and the individual’s ability to critically evaluate digital health content.

**Figure 1:**
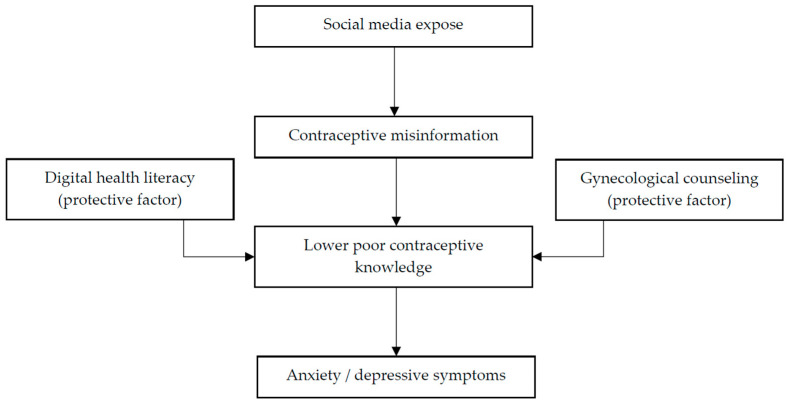
Conceptual framework of social media exposure, contraceptive misinformation, contraceptive knowledge, and psychological outcomes. Proposed conceptual model illustrating the associations identified in the present study between social media use, exposure to contraceptive misinformation, contraceptive knowledge, and psychological well-being among Romanian adolescents and young adults. Digital health literacy and previous gynecological counseling are presented as potential protective factors associated with a lower likelihood of poor contraceptive knowledge. Given the cross-sectional observational design of the study, the framework is intended to illustrate potential relationships observed within the study population and should not be interpreted as evidence of causal or directional pathways. The temporal sequence and directionality of these associations remain uncertain and warrant further investigation in longitudinal studies.

Based on the associations identified throughout the present study, a conceptual framework was developed to illustrate the potential relationship between social media exposure, contraceptive misinformation, contraceptive literacy, and psychological well-being among adolescents and young adults. The proposed model is presented in Figure 1.

## 4. Discussion

The present study provides a nuanced perspective on how Romanian adolescents and young adults navigate contraceptive information in an increasingly digital environment, and more importantly, how this informational landscape translates into both knowledge gaps and measurable psychological outcomes [2]. What emerges from our findings is not simply a shift in where information is obtained, but a deeper transformation in how it is interpreted, internalized, and ultimately integrated into personal health decision-making [4,12].

Before interpreting these findings, it is important to emphasize that the present study employed a cross-sectional observational design. Consequently, the identified relationships should be interpreted as associations rather than causal effects. Although higher misinformation endorsement, lower contraceptive knowledge, and poorer psychological outcomes frequently co-occurred within our cohort, the temporal direction of these relationships cannot be determined. Therefore, the findings should be viewed as reflecting patterns of association within this population rather than evidence that one factor directly causes another.

One of the most striking observations is the overwhelming reliance on social media as the primary source of contraceptive information. While this trend is consistent with broader global patterns, its implications within our cohort appear particularly pronounced. Unlike structured educational settings or clinical encounters, digital platforms operate in an unregulated space where content is driven by engagement rather than accuracy [26]. In this context, adolescents are not merely passive recipients of information, they are active participants in an ecosystem where emotional resonance often outweighs scientific validity. This context may help explain why social media reliance was associated with a higher prevalence of misconceptions within our cohort. However, given the observational nature of the study, it cannot be determined whether social media exposure contributes to these misconceptions or whether individuals with pre-existing misconceptions preferentially seek information from social media sources [27].

Importantly, our data suggest that the issue is not only the presence of misinformation but its disproportionate influence. Participants who reported social media as their main source of information consistently demonstrated lower contraceptive knowledge scores. This inverse relationship highlights a critical vulnerability: exposure to high volumes of information does not equate to understanding. In fact, it may paradoxically contribute to confusion, particularly when conflicting messages are encountered without the necessary framework for critical appraisal. This aligns with the concept of “information overload,” where the sheer quantity of content impairs the ability to distinguish credible from non-credible sources [7,28].

From a clinical perspective, the persistence of specific misconceptions is particularly relevant. The belief that hormonal contraception leads to infertility, reported by over 40% of participants, remains one of the most enduring myths in reproductive health [29]. Its continued prevalence in our cohort suggests that current educational strategies are insufficiently addressing deeply rooted fears, which are often reinforced by anecdotal narratives online. Similarly, the perception that natural methods are equally effective as modern contraception reflects not only a knowledge gap, but also a possible preference for “perceived safety” in the absence of trusted medical guidance.

Beyond the informational dimension, one of the most compelling aspects of this study is the observed association between contraceptive knowledge and mental health indicators. Participants with lower knowledge scores also reported higher levels of anxiety and depressive symptoms. While this association may reflect a relationship between reproductive health literacy and psychological well-being, the cross-sectional design does not allow conclusions regarding directionality. It is equally plausible that psychological distress influences how adolescents seek, process, and interpret contraceptive information [30]. This relationship may be bidirectional. On one hand, uncertainty regarding contraception and exposure to conflicting information may be associated with increased psychological distress. On the other hand, adolescents experiencing higher levels of anxiety may be more likely to search repeatedly for health-related information online, spend more time engaging with reproductive health content on social media, or show greater sensitivity to alarming messages and anecdotal narratives. Consequently, reverse causality cannot be excluded, and the observed associations may reflect a dynamic interaction between information-seeking behavior, misinformation endorsement, and psychological well-being [31,32].

This interplay between knowledge and mental well-being deserves particular attention. It challenges the traditional view of contraceptive education as a purely informational intervention and instead supports a more integrated approach that considers emotional and cognitive factors. In this sense, improving contraceptive literacy may have benefits that extend beyond reducing unintended pregnancies, potentially contributing to better psychological resilience and a greater sense of autonomy [33].

Another important consideration is the relatively limited role of healthcare professionals and formal education in shaping contraceptive knowledge within this population. Despite being the most reliable sources of information, they were among the least frequently reported [34]. This gap raises important questions about accessibility, communication, and trust. It is possible that adolescents perceive clinical environments as intimidating or judgmental, or that opportunities for structured discussions about contraception are simply too limited [35]. Similarly, the modest contribution of school-based education suggests variability in curriculum quality or delivery, which may fail to engage students in a meaningful way.

In the Romanian context, these findings acquire additional relevance. Cultural attitudes toward sexuality, variability in sex education programs, and differences in healthcare accessibility may all contribute to the patterns observed [36]. Compared to Western European settings, where structured sexual education is more consistently implemented, adolescents in Eastern Europe may rely more heavily on informal and digital sources. This makes the quality of online information even more critical, as it often fills the gaps left by formal systems.

From a public health perspective, our results underscore the need for a paradigm shift in how contraceptive education is approached. Rather than attempting to compete with social media, there is an opportunity to leverage these platforms as vehicles for evidence-based information [37]. Interventions that incorporate medically accurate content into formats that are engaging, relatable, and tailored to the digital habits of adolescents may prove more effective than traditional approaches alone. At the same time, strengthening the role of healthcare professionals, through improved communication strategies and youth-friendly services, remains essential [38].

These findings may also have important clinical implications. In everyday practice, reproductive counseling should perhaps no longer focus exclusively on contraceptive methods themselves, but also on the informational environment in which adolescents form their perceptions and decisions. Many young patients are already exposed to large amounts of online content before reaching a medical consultation, and these pre-existing beliefs, fears, and misconceptions may strongly influence both communication and adherence to medical recommendations [39].

In this context, clinicians should consider exploring patients’ primary sources of contraceptive information, particularly their exposure to social media-based content and misinformation [40]. Addressing misconceptions directly during gynecological or adolescent health consultations may help reduce anxiety, improve contraceptive literacy, and strengthen trust in evidence-based medical guidance [41]. At the same time, the findings suggest that digital health literacy should become an increasingly important component of reproductive education strategies [42]. Given the dominant role of social media platforms in this population, integrating medically accurate and accessible educational content into digital environments may represent a more effective approach than traditional educational models alone [43].

The study’s findings should be interpreted considering its observational design. While the associations identified are robust, causality cannot be definitively established. It is plausible that individuals with lower baseline knowledge are more likely to seek information from social media, rather than social media being the primary driver of misinformation. Longitudinal studies would be valuable in clarifying these dynamics. Additionally, self-reported data may be subject to recall or social desirability bias, although the anonymous nature of the survey likely mitigated this to some extent.

### Strengths, Limitations, and Future Directions

One of the main strengths of this study lies in its integrative approach, going beyond a purely descriptive assessment of contraceptive knowledge to explore the complex interaction between information sources, misinformation, and mental health outcomes. By simultaneously evaluating cognitive (knowledge), behavioral (information sources), and psychological (anxiety and depressive symptoms) dimensions, the study provides a more comprehensive understanding of how adolescents engage with reproductive health information in real-world settings. This multidimensional perspective adds depth to the existing literature, which often examines these components in isolation.

Another important strength is the focus on a relatively underrepresented population. Data on contraceptive knowledge and digital health behaviors among adolescents in Eastern Europe, particularly Romania, remain limited. By addressing this gap, the study contributes context-specific insights that may differ significantly from those reported in Western populations, where access to structured sexual education and healthcare services is often more consistent. The relatively large sample size and the inclusion of both behavioral and psychosocial variables further enhance the robustness and relevance of the findings.

In addition, the study captures contemporary patterns of information consumption, particularly the dominant role of social media platforms. This real-world relevance strengthens the applicability of the results, especially in the context of rapidly evolving digital ecosystems. The use of validated instruments for assessing mental health indicators (such as GAD-7 and PHQ-9) also adds methodological rigor and allows for meaningful comparisons with other studies.

However, several limitations should be acknowledged. First, the cross-sectional design inherently limits the ability to infer causality. While significant associations were identified between information sources, knowledge levels, and psychological outcomes, the directionality of these relationships cannot be definitively established. Furthermore, reverse causality represents an important consideration. Adolescents with greater anxiety or health-related concerns may be more inclined to seek information through social media, thereby increasing their exposure to contraceptive misinformation. Longitudinal studies are required to clarify the temporal sequence of these associations and determine whether misinformation endorsement precedes, follows, or mutually interacts with psychological distress. It remains possible, for example, that individuals with lower baseline knowledge or higher anxiety are more likely to rely on social media, rather than social media exposure being the primary driver of misinformation. In addition, the dichotomization of continuous variables such as CKQ and GAD-7 scores may have resulted in some loss of statistical information and reduced power compared with analyses using continuous outcomes. However, these thresholds were selected to facilitate clinical interpretation and to allow identification of participants with relatively lower contraceptive knowledge and clinically relevant anxiety symptoms.

Second, the reliance on self-reported data introduces the potential for recall bias and social desirability bias. Participants may have underreported or overreported certain behaviors, such as contraceptive use or information sources. Although the anonymous nature of the survey likely reduced this risk, it cannot be entirely excluded. Additionally, the assessment of misinformation was based on a predefined set of common contraceptive misconceptions and therefore may not fully capture the breadth, diversity, and rapidly evolving nature of misinformation circulating across digital platforms. The instrument was developed for the purposes of the present study and was intended to reflect frequently encountered misconceptions within the target population rather than to serve as a comprehensive or formally validated measure of contraceptive misinformation. Furthermore, all misinformation items were weighted equally, although certain misconceptions may differ in prevalence, perceived credibility, or potential impact on reproductive health decision-making. Future studies should consider the development and validation of more comprehensive instruments capable of capturing the multidimensional nature of health misinformation in digital environments.

Although the CKQ demonstrated acceptable internal consistency and underwent expert review and pilot testing prior to administration, it has not yet undergone formal external validation in independent populations. Consequently, the findings related to contraceptive knowledge should be interpreted within the context of an exploratory assessment instrument, and future studies should focus on further psychometric validation of the questionnaire.

Another important limitation relates to the sampling strategy and the generalizability of the findings. Participants were recruited using convenience sampling through social media platforms, university student groups, and online peer-sharing networks. Consequently, the study population may not be representative of the broader population of Romanian adolescents and young adults. Individuals who are more digitally engaged, more likely to use social media, and more interested in health-related topics may have been disproportionately represented in the study sample.

In addition, the cohort was predominantly composed of university students and urban residents, which may further limit the applicability of the findings to adolescents and young adults from rural areas, different educational backgrounds, or lower levels of digital engagement. Therefore, the observed associations should be interpreted with caution, and the results should not be considered fully generalizable to all Romanian adolescents and young adults. Future studies employing population-based or nationally representative sampling strategies would help improve external validity and confirm the generalizability of these findings.

In addition, the misinformation score measured endorsement of misinformation-related beliefs rather than objectively verified exposure to misinformation. Therefore, the study cannot determine the specific channels through which these beliefs were acquired, nor the frequency or intensity of participants’ exposure to misinformation.

Furthermore, while the study highlights associations with mental health indicators, it does not explore potential confounding factors in depth. Variables such as family environment, prior education, cultural beliefs, or previous healthcare experiences may influence both knowledge levels and psychological outcomes. Future studies incorporating multivariable analyses or qualitative components could provide a more nuanced understanding of these underlying mechanisms.

In addition, residual confounding cannot be excluded. Although the regression models incorporated several variables considered clinically relevant based on previous literature, other potentially important factors were not available for inclusion. Variables such as parental educational level, family communication regarding sexual health, previous exposure to formal sexual education programs, healthcare access, religious or cultural beliefs, relationship status, and pre-existing psychological vulnerability may influence both contraceptive knowledge and psychological outcomes. Consequently, the observed associations should be interpreted within the context of these potential unmeasured confounders.

Looking ahead, several directions for future research emerge from these findings. Longitudinal studies are needed to better clarify the temporal relationship between misinformation endorsement, knowledge acquisition, and mental health outcomes. Such designs would allow for the identification of causal pathways and critical time points for intervention.

In addition, there is a clear need for intervention-based research. Developing and testing targeted educational strategies, particularly those that integrate evidence-based content into social media platforms, represents a promising avenue. Collaborations between healthcare professionals, educators, and digital content creators could facilitate the dissemination of accurate information in formats that resonate with adolescents.

Expanding research to more diverse populations, including rural communities and younger adolescents, would also be valuable in improving generalizability. Qualitative studies exploring how adolescents perceive and interpret online health information could further enrich our understanding and inform the design of more effective communication strategies.

Future work should consider incorporating measures of health literacy and digital literacy more explicitly, as these constructs likely play a central role in mediating the relationship between information exposure and knowledge outcomes. Addressing these broader competencies may be key to reducing the impact of misinformation and supporting more informed, autonomous reproductive health decisions.

In essence, while the present study provides important insights into the current landscape, it also underscores the need for a more adaptive, multidisciplinary approach to contraceptive education, one that reflects the realities of the digital age and the complex needs of the adolescent population.

## 5. Conclusions

The findings of the present study reflect an important shift in the way Romanian adolescents and young adults access and interpret contraceptive information in the digital era. Social media has become the dominant source of reproductive health information within this population, while traditional educational and medical sources appear to play a considerably smaller role. At the same time, exposure to contraceptive misinformation was highly prevalent and closely associated with lower contraceptive knowledge levels.

More importantly, contraceptive misinformation endorsement and lower contraceptive literacy were associated with higher levels of anxiety and depressive symptoms. These findings suggest a potential relationship between reproductive health literacy and psychological well-being; however, the cross-sectional design does not allow conclusions regarding the directionality or causality of these associations. In addition, multivariable analyses demonstrated that reliance on social media, low digital health literacy, and the absence of prior gynecological counseling were independently associated with poor contraceptive knowledge, while misinformation endorsement and infertility-related fears emerged as significant predictors of anxiety.

Taken together, these findings suggest that social media use, contraceptive knowledge, and psychological well-being were closely interrelated within the study population, with higher contraceptive literacy being associated with more favorable outcomes. However, given the cross-sectional design, the temporal sequence and directionality of these relationships remain uncertain. Further longitudinal studies are needed to determine whether and how these factors influence one another over time.

From a broader perspective, the results underline the need for a more modern and adaptive approach to contraceptive education, one that acknowledges the realities of contemporary digital behavior. Rather than ignoring social media, healthcare professionals and educational systems should actively engage with these platforms and develop evidence-based, accessible, and relatable forms of communication tailored to younger populations.

Ultimately, the present study highlights that contraceptive misinformation endorsement is closely associated with contraceptive knowledge and psychological well-being among Romanian adolescents and young adults. These findings underscore the public health relevance of digital reproductive health information and support the need for further longitudinal and intervention-based research in this area.

## Figures and Tables

**Table 1:** Sociodemographic, behavioral, and digital health characteristics of the study population.

Variable		Total Cohort (*N* = 210)
Age (years) *		19.8 ± 2.1
Urban residence **		131 (62.4%)
University students **		146 (69.5%)
Medical-related education **		38 (18.1%)
Currently sexually active **		114 (54.3%)
Previous contraceptive use **		102 (48.6%)
Previous gynecological counseling **		72 (34.3%)
Smoking **		51 (24.3%)
Alcohol consumption **		63 (30.0%)
Self-perceived socioeconomic status **	Low	39 (18.6%)
	Moderate	132 (62.9%)
	High	39 (18.6%)
Primary source of contraceptive information **	Social media	122 (58.1%)
	School-based education	45 (21.4%)
	Healthcare professionals	31 (14.8%)
	Family/peers	12 (5.7%)
Most frequently used digital platform for health information **	TikTok	88 (41.9%)
	Instagram	56 (26.7%)
	YouTube	31 (14.8%)
	Online forums/blogs	21 (10.0%)
	Other	14 (6.7%)
Time spent consuming health-related content on social media **	<1 h/day	62 (29.5%)
	1–3 h/day	97 (46.2%)
	>3 h/day	51 (24.3%)
Main contraceptive method previously used **	Male condom	61 (29.0%)
	Combined oral contraceptives	27 (12.9%)
	Emergency contraception	21 (10.0%)
	Natural methods	18 (8.6%)
	No prior contraceptive use	83 (39.5%)
Most common contraceptive-related fear **	Infertility	74 (35.2%)
	Weight gain	49 (23.3%)
	Hormonal imbalance	42 (20.0%)
	Thrombotic risk	28 (13.3%)
	Depression/mood changes	17 (8.1%)
Digital health literacy level **	Low	58 (27.6%)
	Moderate	103 (49.0%)
	High	49 (23.3%)
Mean CKQ *		6.8 ± 2.1
Mean GAD-7 *		7.1 ± 4.3
Mean PHQ-9 *		6.4 ± 4.1

Data are presented as mean ± standard deviation (SD) for continuous variables (*) and as number (percentage) for categorical variables (**). CKQ = Contraceptive Knowledge Questionnaire; GAD-7 = Generalized Anxiety Disorder-7 scale; PHQ-9 = Patient Health Questionnaire-9. Higher CKQ scores indicate better contraceptive knowledge, while higher GAD-7 and PHQ-9 scores reflect greater severity of anxiety and depressive symptoms, respectively. Digital health literacy levels were categorized based on cumulative questionnaire scores as low, moderate, or high.

**Table 2:** Primary sources of contraceptive information.

Source of Information	*n* (%)
Social media	122 (58.1%)
School-based education	45 (21.4%)
Healthcare professionals	31 (14.8%)
Family/peers	12 (5.7%)

Data are presented as number (percentage) of participants reporting each source as their predominant source of contraceptive information. Social media platforms included digital applications frequently used for health-related content consumption, whereas healthcare professionals included physicians, gynecologists, nurses, and other medical personnel involved in reproductive health counseling.

**Table 3:** Prevalence of common contraceptive misconceptions.

Misconception	*n* (%)
Hormonal contraception causes infertility	87 (41.4%)
Natural methods are as effective as modern methods	80 (38.1%)
Contraceptives severely affect long-term health	70 (33.3%)
Emergency contraception is abortive	60 (28.6%)

Data are presented as number (percentage) of participants endorsing each misconception related to contraception and reproductive health. Misconceptions were assessed using predefined questionnaire items reflecting commonly encountered inaccurate beliefs regarding contraceptive safety, effectiveness, fertility, and emergency contraception. Participants could endorse more than one misconception.

**Table 4:** Correlation between information source, knowledge, and mental health indicators.

Variable	CKQ Score	GAD-7 Score	PHQ-9 Score
Social media use	−0.31 *	0.42 **	0.38 **
Medical information	0.36 *	−0.28 *	−0.25 *
Misinformation endorsement	−0.44 **	0.47 **	0.41 **

Values represent Pearson correlation coefficients (r) describing the associations between information-related variables and psychological or knowledge-based outcomes. CKQ = Contraceptive Knowledge Questionnaire; GAD-7 = Generalized Anxiety Disorder-7 scale; PHQ-9 = Patient Health Questionnaire-9. Positive coefficients indicate direct associations, whereas negative coefficients indicate inverse associations. * *p* < 0.05; ** *p* < 0.001.

**Table 5:** Multivariable logistic regression model for predictors of low contraceptive knowledge.

Variable	OR	95% CI	*p*-Value
Primary reliance on social media	2.21	1.12–4.34	0.022
Predominant TikTok use	1.58	0.89–2.81	0.116
Low digital health literacy	2.94	1.51–5.71	0.001
No prior gynecological counseling	1.87	0.98–3.58	0.058
Rural residence	1.33	0.71–2.49	0.372
Low socioeconomic status	1.69	0.88–3.23	0.112
Non-medical education	2.41	1.01–5.74	0.047

Odds ratios (ORs) with 95% confidence intervals (CIs) were calculated using multivariable logistic regression analysis. Low contraceptive knowledge was defined as a CKQ score below the cohort median (CKQ < 7). The regression model included demographic, educational, and digital behavioral variables considered clinically relevant based on previous literature. OR values > 1 indicate increased odds of low contraceptive knowledge. Statistically significant associations were considered at *p* < 0.05.

**Table 6:** Multivariable logistic regression model for predictors of moderate-to-severe anxiety.

Variable	OR	95% CI	*p*-Value
High misinformation endorsement	2.36	1.28–4.35	0.006
Low contraceptive knowledge	2.11	1.14–3.91	0.018
Predominant TikTok use	1.62	0.91–2.89	0.099
High health-related social media exposure (>3 h/day)	1.98	1.04–3.76	0.037
Infertility-related contraceptive fear	2.54	1.32–4.88	0.005
Low digital health literacy	2.27	1.19–4.31	0.013

Odds ratios (ORs) with 95% confidence intervals (CIs) were calculated using multivariable logistic regression analysis. Moderate-to-severe anxiety was defined as a GAD-7 score ≥ 10. The regression model included variables related to contraceptive misinformation endorsement, digital behavior, contraceptive knowledge, and perceived reproductive concerns. OR values > 1 indicate increased odds of moderate-to-severe anxiety. Statistical significance was established at *p* < 0.05.

## Data Availability

The data presented in this study are available upon request from the corresponding author. The data are not publicly available due to privacy restrictions.
